# Olfaction, valuation, and action: reorienting perception

**DOI:** 10.3389/fpsyg.2014.00299

**Published:** 2014-04-08

**Authors:** Jason B. Castro, William P. Seeley

**Affiliations:** ^1^Psychology and Neuroscience, Bates CollegeLewiston, ME, USA; ^2^Philosophy, Bates CollegeLewiston, ME, USA

**Keywords:** piriform cortex, value encoding, olfactory bulb model, affective neuroscience, hedonic valence, categorical perception, object perception

## Abstract

In the philosophy of perception, olfaction is the perennial problem child, presenting a range of difficulties to those seeking to define its proper referents, and its phenomenological content. Here, we argue that many of these difficulties can be resolved by recognizing the object-like representation of odors in the brain, and by postulating that the basic objects of olfaction are best defined by their biological value to the organism, rather than physicochemical dimensions of stimuli. Building on this organism-centered account, we speculate that the phenomenological space of olfaction is organized into a number of coarse affective dimensions that apply categorically. This organization may be especially useful for coupling sensation to decision making and instrumental action in a sensory modality where the stimulus space is especially complex and high dimensional.

Describing the phenomenology of smells is notoriously difficult. Why is this? One idea is that odor percepts are “impoverished,” as they are initially processed by phylogenetically older parts of the brain via a shallow processing stream, with no obligatory relay in the thalamus. By this logic, olfaction is presumed to be something like visual sensation in cortically blind individuals: there is some basic stimulus awareness, but stimuli simply are not perceived in a feature-rich way that provides grist for analysis and description. One might, alternatively, interpret the putative computational shortcomings of olfaction as artifacts of the representational problem to which the olfactory system is addressed. Visual and auditory processing transform topographically encoded physical quantities into representations of stable object properties that facilitate physically interactive behavior like object recognition, reaching, grasping, orienting, or avoiding. Olfactory processing, on the other hand, begins with a diverse, high dimensional, and niche-specific set of physicochemical stimuli (Bargmann, 2006), and yields affective responses that facilitate the evaluation of the biological significance of stimuli. These object features and phenomenological responses don’t sort easily into a clear metric for organizing and understanding either the physicochemical space of odorants or the phenomenal space of olfactory experience. The conjunction of these facts about the disorderly stimulus space and phenomenology of olfaction has led some philosophers to question whether olfactory percepts have any representational content at all (see Batty, forthcoming):

“Smell has little in the way of apparent structure and often floats free of any apparent object, remaining a primitive presence in our sensory manifold”

(Chalmers, 1996)

“Phenomenologically speaking, a smell is just a modification of our consciousness, a qualitative condition or event in us”

(Lycan, 2000)

“a sensation of [smell]…may have no representational content of any sort, though of course the sensation will be of a distinctive kind”

(Peacocke, 1983)

Recent research suggests that philosophical skepticism about the representational capacities of olfactory perception is a straw man, and more importantly, perhaps beside the point. Stimulus representation isn’t the primary business of olfaction. Rather, its job is solving a problem of valuation, rapidly encoding the biological salience of a stimulus and priming our multisensory representation of it to contextually appropriate action.

We develop our perspective in two parts. We first review physiological and functional imaging studies showing that it is quite appropriate to regard odors as objects – that is, as perceptual phenomena bearing most of the hallmarks of object-based processing. In doing this, we underscore the critical idea that the “feature-poor” nature of odor objects is not a bug, but rather an important computational feature of the system tagged to the function of olfactory representations in the broader cognitive economy of human perceptual systems. In fact, the olfactory system is amply equipped to represent information about discrete molecular features, yet actively discards or reformats these representations in favor of more economical or parsimonious representations. In the second part of our perspective, we speculate on what these representations might be. In brief, we suggest that the objects of olfaction consist of coarsely specified and categorical affective dimensions that each serve as an invitation to a prescribed kind of action or consummatory behavior. By “affective,” we mean to designate bodily states that carry information about the biological value of a stimulus, and that serve as the foundation for approach and withdrawal behaviors. We propose that a given odor percept is a coordinate point along one of these dimensions, and furthermore that these dimensions are best defined from the perspective of the organism in an ecological context. This “reorienting” of perception may help develop a satisfying philosophical stance on olfaction that also provides impetus for physiological study.

## ODORS AS OBJECTS

Philosophical skepticism about the representational capacities of olfaction arises as a question about the nature of olfactory percepts: do smells represent discrete, publically shareable perceptible objects as visual percepts do or are they more akin to subjective feelings, affective states that carry interoceptive information about bodily state? We think that this distinction is ill-formed. For instance, a range of recent studies demonstrate that many of the hallmarks of object-based visual processing readily apply to olfaction. We can, for example, segregate an odor from its surround, recognize discrete, non-overlapping “views” as representations of the same odor object, categorize different odors as exemplars of the same type, and discriminate individual stimuli within categories of odor objects (Stevenson and Wilson, 2007; Gottfried, 2010; and references therein). Critically, however, the chemosensory features that support this kind of object-based processing are unavailable to conscious awareness. The olfactory system therefore extracts information about the ensemble of structural features in a molecule and uses it to discriminate and identify smells in the local environment, as this information is readily encoded in bulbar and early cortical representations of the anterior piriform cortex (APC) (Gottfried et al., 2006; Kadohisa and Wilson, 2006). However, and this is the real rub, the olfactory system ultimately reformats this stimulus information into a range of affective dimensions efficiently tuned to the behavioral needs of the perceiver.

In this regard, it is interesting to consider the different neural codes employed to encode stimulus information by the APC vs. the posterior piriform cortex (PPC). To broadly summarize a number of studies (see Gottfried, 2010), the distributed patterns of odor-evoked activity in APC encode a snapshot of the composite sum of an odor’s constituent features as a configural cue. Over time, the connectivity that defines these distributed activation patterns is reinforced and comes to serve as a template, or a cue-dependent, content addressable memory of the constituent image of that odor. In this context a novel view or degraded stimulus might contain sufficient information to activate a memory representation of the target odorant, accounting for perceptual constancy across sniffs and natural ecological variance.

Neural codes in the PPC, in contrast, may encode qualitative perceptual similarities and differences among individual odorants, facilitating the construction of common odor categories that are called upon in categorization and discrimination tasks. Although the distributions of activity for odors differing in perceptual quality are spatially diffuse and overlapping like their APC cousins, multi-voxel pattern analysis (which is sensitive to the particular distribution of activity) in PPC demonstrates that qualitatively distinct odorants elicit unique patterns of activation. Notably, the degree of overlap among qualitatively similar odorants is higher than for dissimilar odorants, and voxel-wise patterns can be used to accurately predict category membership for a given stimulus (Howard et al., 2009). The quality space of conscious olfactory perception thus seems well-modeled as a value space defined by the relative pleasantness of odorants, and shaped in part by associative learning. In this regard, the object information carried in conscious olfactory events is information about the significance of odor sources to our apical and instrumental goals.

## ODOR ECOLOGY

As many have noted (Yeshurun and Sobel, 2010), and intuition will confirm, it is trivially easy to report whether one likes a given smell. Notably, this “readiness” to like or dislike doesn’t extend to visual stimuli, suggesting there may be range of tacit value judgments that are intrinsic to olfactory objects. Thinking about these phenomenological differences in light of the distinct ecological contexts in which vision versus olfaction predominate can be instructive. Visual objects are sensorimotor representations of stimulus structure that articulate the shape, identity and affordances of objects and events so that we can recognize them, orient our bodies to them and interact with them. Simply stated, fine grained shape information is what we need to accomplish these tasks. An olfactory object, we would argue, is a qualitative judgment of the biological significance of the odor source, its utility to our metabolic needs and the apical goal of survival – an implicit decision about whether something is worth approaching, or is best avoided.

To early single-celled denizens of the pre-Cambrian, counting double-bonds and tallying functional groups on a molecule were probably not useful in themselves. Rather, they were likely only useful to the extent that they led to good decisions about whether toxins, nutrients, and discrete signals from conspecifics were fled, pursued, ingested, or prompted consummatory behavior. An upshot of this view is that it readily explains those well-known cases in which chemicals with quite different structures elicit highly similar percepts (their physical differences aside, they happen to point to the same biological need/want), and vice versa – chemicals that are close “neighbors” in physicochemical space may elicit very different percepts (their physical similarities aside, one is a toxin, say, whereas the other is a nutrient). By carving the phenomenal space of olfaction into a number of prescribed affective categories (which can be plied substantially by experience), evolution may have ensured that olfactory perception is intimately coupled to stimulus-prompted decision making.

## THE PRIMACY OF AFFECT, AND ITS MANIFESTATION IN OLFACTORY BRAIN AREAS

The primacy of affect in olfactory experience has long been appreciated, and recent work applying dimensionality reduction techniques to odor profiling databases underscores this basic idea. Khan et al. (2007) and Zarzo (2011) used principal components analysis to identify hedonic valence as a factor accounting for about 40% of the variance in olfactory percepts when a wide range of odors is assessed. Intriguingly, when similar dimensionality reduction techniques were applied independently to the physicochemical space of odors, the major axis of this space – something like “molecular compactness” – was mapped onto hedonic valence. In a sense then, pleasantness is “written into” a monomolecular odorant. In studies extending these sorts of analyses above, a second (though much more speculative) candidate dimension – “edibility” – has been postulated (Zarzo, 2008). We note that many other candidate dimensions have been proposed by others as well, however, the existence and commonality of these is contested.

Consistent with this claim on the primacy of affect, affect has interesting correlates even in the most peripheral stages of olfactory processing. Whereas the receptor epithelia for vision, audition, and somatosensation are topographically organized to encode information about spatial proximity and/or basic physical variables pertaining to stimuli, the (human) olfactory epithelium appears to represent relative stimulus pleasantness topographically (Lapid et al., 2011). Similar principles of organization by hedonic valence also seem to extend to early central brain representations in both mammals and invertebrates (Haddad et al., 2010).

Building off of this work, we speculate that the affective (hedonic) dimension of olfactory experience is fundamental, and that olfactory categories, as a result, carry information about the behavioral salience of their sources as opposed to the specific identities of either the odorant perceived or its source (see also Mamlouk et al., 2003). We are not claiming that the olfactory system fails to map those features of the sensory periphery that facilitate object discrimination, recognition, etc., Indeed, certain elemental features of monomolecular odor stimuli, including information about constituent functional groups, are readily encoded and available to the olfactory system, as seen above. However, we propose that they are encoded in conscious olfactory experience as a fixed (if potentially quite large) number of coarsely specified affective dimensions. Put more plainly: the olfactory system is not a casual art viewer, who slowly scans the individual brushstrokes on a painting and dithers on whether these add up to something he likes or doesn’t. The olfactory system is instead a curmudgeonly and narrow-minded critic who knows he loves Picasso, despises Monet, is indifferent to Kandinsky, and rapidly judges the value of a work by its resemblance to one of these categories.

While pleasantness is undisputably a key organizing dimension of olfaction, and the one that currently has the deepest experimental support, it needn’t be the *only* dimension. Organisms may have multiple ways of evaluating odors as good, and multiple ways of evaluating them as bad. For example, floral odors may have one set of affordances (approach, but do not necessarily consume), whereas odors comparable in pleasantness – say citrus odors – may have another set of affordances (approach and consume). In short, olfactory percepts may be defined by multiple categorical affective dimensions, rather than a single smoothly varying dimension.

In our own studies, we recently revisited the issue of perceptual organization in odor profiling data, using non-negative matrix factorization (NMF) instead of principal components analysis, and observed that odor profiles are well described by ~10 perceptual dimensions (Castro et al., 2013). Notably, these dimensions were near-orthogonal, despite the fact that orthogonality is not guaranteed by NMF, and appeared to apply categorically: that is, a given odor tended to belong to one perceptual dimension to the *exclusion* of others. Interestingly, the basic dimensions identified all seemed to have clear ecological value. Future work will be needed to extend these findings to larger panels of odorants, and to test whether there are neural substrates of the coarse affective categorization we propose.

Nevertheless, these sorts of either-or representations may have some observable analogs in olfactory physiology. The possible categorical nature of odor representations is supported by studies performing slow “morphs” between two different odors, in which the concentration of one odor is gradually decreased, while the other is slowly ramped up. In these studies, one observes rapid changes in ensemble activity in the olfactory bulb (Niessing and Friedrich, 2010), implying that at least for some considerations of odor pairs, the bulb has a fixed number of preferred discrete states, rather than smoothly spanning the potential state space. More centrally, at the level of piriform cortex, Yoshida and Mori (2007) have observed categorical representations for food odors in the APC, whereas Howard et al. (2009) have used multi-voxel analysis in human fMRI studies to show clustered hotspots of activity in PPC that correspond to specific odor qualities (minty, woody, citrus, etc).

## SUMMARY AND CONCLUSION

By summarizing recent work on object-based processing in olfaction, as well as odor ecology, we have argued that the basic phenomenological objects of olfaction are not things “out there” but rather prescribed affective categories – likely niche and organism specific – to which stimuli are rapidly assigned, and which are richly pliable through learning and with context. We are quick to note, of course, that we are *not* arguing that odor stimuli are irrelevant to this categorization process. Our account is necessarily speculative, but aligns in several compelling ways with existing results, and makes predictions about the types of representations one expects to find in the olfactory system. Contrary to some of the modern philosophical thinking on olfaction, we speculate that a careful and exhaustive cataloging of behaviors supported by olfaction may be a key to understanding the phenomenology of odors.

## Conflict of Interest Statement

The authors declare that the research was conducted in the absence of any commercial or financial relationships that could be construed as a potential conflict of interest.
